# Evaluation of effectiveness of (elements of) parenting support in daily practice of preventive youth health care; design of a naturalistic effect evaluation in ‘CIKEO’ (consortium integration knowledge promotion effectiveness of parenting interventions)

**DOI:** 10.1186/s12889-019-7785-y

**Published:** 2019-11-06

**Authors:** Dafna A. Windhorst, Yuan Fang, Irene N. Fierloos, Matty R. Crone, Krista Van Mourik, Harrie Jonkman, Clemens M. H. Hosman, Wilma Jansen, Hein Raat

**Affiliations:** 1 Department of Public Health, Erasmus University Medical Center, Rotterdam, the Netherlands; 2 Department of Public Health and Primary Care, Leiden University Medical Center, Leiden, The Netherlands; 3 Verwey-Jonker Institute, Utrecht, the Netherlands; 4 Department of Health Promotion, Maastricht University, Maastricht, the Netherlands; 5 Department of Clinical Psychology, Radboud University, Nijmegen, the Netherlands; 6 Hosman Prevention and Innovation Consultancy, Bergen Dal, the Netherlands; 7 Municipality of Rotterdam, Rotterdam, the Netherlands

**Keywords:** Effective elements, Intervention, Naturalistic effect evaluation study, Parenting, Design

## Abstract

**Background:**

The number of interventions to support parents is growing. The level of evidence regarding these intervention varies. In this paper we describe a study that aims to assess the effectiveness of specific ‘elements’ within such parenting interventions for families with children up to 7 years. A naturalistic effect evaluation will be applied. Study questions are:

1. What is the exposure of parents to (elements of) parenting interventions in the daily practice of preventive youth health care?
2. What are the associations between the exposure to (elements of) parenting interventions and outcomes in parents/children related to parenting and child development?

**Methods/design:**

Thousand parents/caregivers are recruited by preventive youth health care providers in the Netherlands. Measurements will be performed after inclusion and after 12-months follow up. Data regarding child/parent/caregiver characteristics, use of (parenting) interventions and care, and outcomes with regard to parenting skills, family functioning and child development will be collected. Outcomes will be compared between parents/children exposed and non-exposed to the (elements of) parenting interventions (adjusting for confounders).

**Discussion:**

We hypothesize that parents/caregivers with exposure to (elements of) parenting interventions show (relatively more) improvements in parenting outcomes. Results will support intervention selection/development, and support communities/professionals to select appropriate intervention-elements.

**Trial registration:**

Netherlands National Trial Register number NL7342. Date of registration: 05-November-2018, retrospectively registered.

## Background

Parental concerns are highly prevalent in the general population, and can therefore be considered as a usual aspect of parenting. In the Netherlands, studies report up to 50% of parents who have questions or concerns regarding the upbringing of their child [1–3]. Examples of topics of concern are child development, child health and diseases, children’s (difficult and disobedient) behavior, socio-emotional development of children and general parenting (skills) [1, 4]. Many parents feel the need to discuss their concerns with professionals or to receive other forms of parenting support [1].

Parenting support can be described as all activities, services, programs and interventions aimed at increasing parenting skills and improving the upbringing situation [1, 5]. There are various sources of parenting support, varying from informal support provided by one’s own social network, to services provided by semi-formal support sources (e.g. community-based voluntary sector organizations) or by formal support sources (e.g. typically involving statutory sector providers, or statutory and voluntary sector partnerships) [6]. Activities and interventions can be ‘universal’, aimed at all parents/caregivers, or ‘targeted’ at specific groups of parents who experience mild to severe levels of parenting problems or parenting stress, or who report behavior problems of their children [7].

Over the years, numerous parenting interventions have been developed with varying content, delivery settings, delivery techniques, types of families served and intended outcomes [8]. In general, there is considerable evidence that parenting interventions can have positive effects on a range of parent and child outcomes including parenting skills, parents’ self-efficacy and self-esteem, parent’s psychosocial health, parenting stress and child behavior [7–11].

The number of parenting interventions continues to grow. However, the level of the evidence regarding the effectiveness of the interventions varies. This makes it difficult for professionals to select the most appropriate and effective interventions. Moreover, studies investigating effectiveness of interventions are often focused on the interventions as a whole, while interventions generally consist of many elements. In order to better understand effects and mechanisms of behavior change interventions, it is important to identify which elements of the interventions contribute to the effects [12, 13]. These insights will be helpful for the development of new interventions and for intervention selection. Furthermore, it could enhance broader and more efficient implementation of existing interventions if these can be adapted to local needs and opportunities, and to the needs and strengths of the clients.

So, in addition to knowledge regarding the effectiveness of interventions, knowledge on the effectiveness of elements within the interventions is needed; but still scarce. Only a few meta-analyses that consider elements in parenting interventions have been carried out [8, 14, 15]. Therefore, the CIKEO consortium (Consortium Integration Knowledge promotion Effectiveness Of parenting interventions) aims to investigate effectiveness of elements of preventive parenting support. Knowledge is obtained from multiple sources: from published scientific research, professionals and families. In this paper we discuss the study protocol of a naturalistic effect evaluation within CIKEO to evaluate the effectiveness of parenting interventions in the daily practice of preventive youth health care (see below). The study aims to provide insight in both the effectiveness of interventions, as well as of elements of parenting support. For the latter, a relevant taxonomy of elements of parenting support will be applied (see below).

The CIKEO consortium is one of six consortia that are funded by ZonMw, the Netherlands Organization for Health Research and Development, in order to investigate the effectiveness of elements of all the interventions that are targeted to youth and families and that are registered in the national database of effective youth interventions in the Netherlands (Databank Effectieve Jeugdinterventies, website: www.nji.nl/nl/Databank/Databank-Effectieve-Jeugdinterventies)). Each consortium is focused on a different theme of interventions. The CIKEO consortium is a collaboration between partners of three Academic Collaborative Centrers (in Dutch: Academische Werkplaatsen) from the regions of Rotterdam, Leiden and Amsterdam in the Netherlands. Academic Collaborative Centers are local collaborations of academic institutions, public health services, municipal policy makers, elected officials and other relevant sectors. Knowledge exchange between these parties can stimulate the translation of scientific knowledge into practical products, services and facilities [16–18]. The executive partners of the CIKEO consortium are the Erasmus University Medical Center in Rotterdam, the Leiden University Medical Center in Leiden and the Verwey-Jonker Institute in Utrecht.

The CIKEO consortium started with an inventory study to give a brief up-to-date overview of the needs of parents, the use of preventive parenting interventions and the existing knowledge regarding the effectiveness of these interventions. The CIKEO consortium investigates parenting support using the approach of a naturalistic effect evaluation [19, 20] with a focus on the distinct elements within parenting interventions, and the ways the interventions are delivered. The elements of the parenting interventions are categorized according to the behavior change technique that are being applied, as proposed by Michie et al. [12]; this is referred to the “taxonomy of Michie” [12].

In this paper, the design and methods of the naturalistic effect evaluation [19] of the CIKEO consortium will be described in detail. This is a prospective cohort study among parents with children up to 7 years. The objectives of the study are:

1. To study the exposure of parents to (elements of) parenting interventions and other types of parenting support in the daily practice of the preventive youth health care.
2. To investigate the associations between the exposure to (elements of) parenting interventions and other types of parenting support and outcomes in parents and children related to parenting and child development (see below).

The hypothesis is that parents/caregivers who receive (elements of) parenting support during the follow-up period will show (relatively more) improvements in parenting skills, family functioning and child development compared to comparable parents/children who did not receive parenting interventions.

## Methods/Design

### Preventive youth health care in the Netherlands

The preventive youth health care is a system for monitoring children’s health and development, and for providing health promotion and disease prevention [21]. It is offered nation-wide and free of charge, independent of insurance status. Participation is voluntary. From birth onwards, parents are frequently invited with their child to visit preventive youth health care centers. The attendance rate is about 95%. During these visits, the growth, development, health and well-being of the child are assessed [21–23]. Additionally, youth health care professionals provide information about the development and upbringing of children and promote a stimulating pedagogical climate. They aim to identify child behavior problems or parenting problems in an early stage and organize help when needed. The youth health care professionals can provide pedagogical counseling or they can direct parents and families to parenting interventions offered by themselves or other providers [1, 24]. In this way, youth health care providers can serve as link with specialized services; they are the frontline services for a broad range of questions [24]. Municipalities are responsible for the preventive youth health care and facilitate a suitable offer of preventive services and coordinate the collaboration between these services [1, 24].

### Parenting interventions and parenting support

For this study, parenting interventions of interest are defined as follows: Interventions for parents with children aged up to 7 years old, targeted at improving parenting skills, parental competences, child development and behavior of the child. The interventions in this study have the aim to prevent (parenting) problems to arise or to intervene with high risk groups or in families with mild problems, to prevent problem from becoming more severe. Interventions for treatment of severe or clinical problems, are beyond the scope of this study.

The CIKEO consortium will study a selection of 21 preventive interventions (see Additional file 1), based on national professional youth health care guidelines [1] and on inclusion within the Dutch national Database of Effective Youth Interventions (in Dutch: Databank Effectieve Jeugdinterventies) of the Netherlands Youth Institute (www.nji.nl/nl/Databank/Databank-Effectieve-Jeugdinterventies) in the year of 2017. In order to be eligible for inclusion in this database, interventions should have a structured and goal-oriented approach, aimed at children and youngsters aged -9 months to 24 years, their caregivers and or parenting environment. The intervention should be carried out in the Netherlands and should have an accessible manual in Dutch language. At least a limited process evaluation should be available. Finally, the intervention should be submitted by the owner or licensee. Once these criteria are met, the intervention is further assessed by an independent committee of national experts to be accepted in the database and to be classified into one of four categories differing in indications of effectiveness [25].

Of the CIKEO selection of 21 interventions, 19 interventions are relevant to the population of this study; parents/caregivers of children up to 7 years old (see Additional file 1). In addition, this study also includes a) practice-based interventions that are not registered in the database of effective youth interventions, b) consultations regarding parenting, c) informal types of parenting support (for example parenting books, websites) and d) regular monitoring visits to the youth health care centers.

### Study design

The design is a naturalistic effect evaluation [19]. It is an observational prospective cohort study with a baseline and follow-up measurement (12 months after inclusion). The total study sample will consist of parents with various levels of parenting problems and concerns. Both at baseline and at follow-up, all participating parents/caregivers will be asked to complete a questionnaire. There are no separate intervention and control groups in this study. The exposure of parents/caregivers to (elements of) parenting support during the follow-up period will be established by the follow-up questionnaire and from electronic files of the youth health care organizations (only with permission by the study participants, see below). By comparing the outcome measures between baseline and follow-up measurements, we aim to investigate which interventions and/or elements of parenting support are associated with a relative decrease in problems and improvement in positive outcome measures in parents and their children.

Data collection started in October 2017 and will continue until April 2020.

### Ethics

The research proposal was reviewed by the Medical Ethics Committee of the Erasmus Medical Center, Rotterdam. Based on their review, the Committee concluded that the rules laid down in the Dutch Medical Research Involving Human Subjects Act (also known by the Dutch abbreviation WMO, in full ‘Wet Medisch-wetenschappelijk Onderzoek met mensen’) do not apply to this research proposal (proposal number MEC-2017-432), and gave permission to conduct this study at Erasmus Medical Center and to submit the results for publication in a scientific journal in the future (Letter NL/sl/321518; 24/07/2017). All participants in the study will provide written informed consent.

### Participants and recruitment

We aim to include a total of net 1000 families in the cohort study. Recruitment will take place in two parts.

Part A) A sample of net 800 parents/caregivers of children aged 0–7 year will be included by two regional preventive youth health care providers (CJG Rijnmond and Rivas Zorggroep) in the regions of Rotterdam and Dordrecht, the Netherlands. These preventive youth health care organizations will inform a sample of parents/caregivers of children aged between 15 and 21 months and children aged 5 or 6 years (second grade of primary school) from their registries about the study, and invite them to participate. All invited parents/caregivers will receive project information, an informed consent form and a baseline questionnaire. Parents/caregivers who are willing to participate are requested to return the completed baseline questionnaire and the signed informed consent form to the researchers in a pre-paid envelope or via internet. All parents/caregivers who provide informed consent and a completed questionnaire are enrolled in the study.

Part B) Part B concerns an opportunity sample of net 200 parents/caregivers of children aged 0–7 year who will participate in a parenting intervention during the follow-up period. Preventive youth health care organizations and other providers of parenting support are informed about the study. They are asked to inform parents who participate in interventions about the study. The procedure regarding study information, informed consent and data collection is similar as for the parents in part A. In addition, participants are recruited directly through advertisements on websites about parenting. They will be directed to the digital version of the project information, the informed consent form and the questionnaire. All parents/caregivers who provide informed consent and a completed questionnaire are enrolled in the study.

### Data collection and measurements

Data will be collected through questionnaires that will be filled out by the primary caregiver of the child at baseline (time of inclusion) and after a follow-up period of twelve months. The questionnaires can be completed on paper or digital through a secured website. All questionnaires consist of valid and evidence-based instruments; details are described below. In the questionnaire, the participants can indicate whether they want support by a professional caregiver; if so, they will be referred accordingly.

### Outcome measures

The outcomes of this study are variables related to parenting, family life and child development. Unless otherwise specified, measures will be assessed at baseline as well as at follow-up.

General parenting styles and parental practices will be measured using two scales previously described by Wake [26]. The sub scale ‘warmth’ comprises six items from the Child Rearing Questionnaire [27] addressing the frequency of warm affectionate behaviors of parents towards their children. The sub scale ‘control’ includes five items from the National Longitudinal Survey of Children and Youth (Statistics Canada. National Longitudinal Survey of Children), addressing the frequency with which parents set and enforce clear expectations and limits. Items will be rated on 5-point scales, ranging from 1 (never/almost never) to 5 (all the time). The warmth and control sub scales will be used to define four parenting styles: authoritative (high warmth and high control), authoritarian (low warmth and high control), permissive (high warmth and low control), and disengaged (low warmth and low control).

Dysfunctional discipline strategies in parents will be measured with the Parenting Scale (PS) [28]. The PS consists of 30 items. Each item is rated on a seven-point Likert scale, ranging from a high probability to use an effective discipline strategy to a high probability of using ineffective discipline strategies. One item (When my child misbehaves, I spank, slap, grab, or hit my child) will not be assessed in this study, due to perceived invasiveness to parent(s)/caregiver(s). Three subscales will be computed: over-reactivity, laxness and verbosity.

Daily parenting stress will be measured with the Parenting Daily Hassles Scale (PDH) [29, 30]. The measure consists of 20 typical daily events in parenting and parent-child interactions. Parents/caregivers will be asked to rate the frequency of each potential hassle in the previous week on a 4-point scale (rarely, sometimes, a lot, constantly). Additionally, they will rate the intensity of the hassles on a 5-point scale, ranging from 0 (no burden) to 4 (great burden).

Parents’/caregivers’ satisfaction with parenting and their self-efficacy in the parenting role will be assessed with the Parenting Sense of Competence Scale (PSOC) [31]. The PSOC contains 17 items with two subscales: satisfaction and efficacy. Parents indicate their agreement with a series of statements regarding degree of confidence and satisfaction in carrying out their parenting role on a 6-point Likert scale (1 = strongly agree, 6 = strongly disagree). Higher scores indicate higher levels of parenting self-efficacy and satisfaction.

Family stress will be assessed by the seventh subscale (General Functioning) of the Family Assessment Device (FAD) [32, 33]. The FAD is a self-report measure of family functioning. The General Functioning scale consists of 12 items about support and stress within the family, providing a measure of overall health/pathology of the family. Each item receives a score from 1 (strongly agree) to 4 (strongly disagree), with the scale for the negatively worded items reversed. The sum of values is divided by 12 to give a total score ranging from 1 to 4. Higher scores indicate poorer family functioning.

Children’s emotional and behavioral problems will be assessed using the Child Behavior Check List (CBCL/1,5–5) [34]. The CBCL contains 99 items concerning the child’s behavior in the previous 2 months. Each item is scored on a three-point scale with 0 (not true), 1 (somewhat or sometimes true) and 2 (very true or often true). The CBCL includes seven empirically-based syndrome scales: emotionally reactive, anxious/depressed, somatic complaints, withdrawn, sleep problems, attention problems, and aggressive behavior. Two broad band scales can be derived. An internalizing problem score can be derived by summing the subscales emotionally reactive, anxious/depressed, somatic complains, and withdrawn and an externalizing problem score can be derived by summing the subscales attention problems and aggressive behavior scales. A total problem score can be computed by summing all 99 items. Additionally, there are five scales oriented at the Diagnostic and Statistical Manual for Mental Disorders (DSM); affective problems, anxiety problems, pervasive developmental problems, attention deficit/hyperactive problems, and oppositional defiant problems. Higher scores indicate higher levels of emotional and behavioral problems.

Other outcomes are sleep, eat and cry behaviors of the child. Parents’/caregivers’ ratings of the prevalence of problems and their concerns regarding these behaviors are assessed with 9 items for sleep behaviors, 5 items for eat behaviors and 2 items for cry behaviors.

### Other measures

Characteristics of the parents/caregivers including age, gender, country of birth, educational level, employment situation and marital status are collected. Perceived social support, general happiness, general health and psychological distress are also assessed. Social support perceived by parents/caregivers will be measured using the Multidimensional Scale of Perceived Social Support (MSPSS) [35, 36]. Happiness will be measured with a single item “Do you feel happy in general” on an 11-point scale [37]. General health will be measured using the first item of the short form 12 (SF-12) health survey [38]. Psychological distress will be measured using the Brief Symptom Inventory 18 (BSI-18) [39]. The BSI-18 includes three subscales: depression, anxiety, and somatization. Additionally, the global severity index will be computed as an overall general psychological distress score. One item (thoughts of ending your life) will not be assessed in this study, due to possible invasiveness to the parent/caregiver.

Characteristics of the child include gender, age, country of birth, birth weight, gestational age at birth, pregnancy and birth complications, general health (assessed with the first item of the Child Health Questionnaire [40], use of child daycare service, medicine use for problems regarding social and emotional development and diagnoses of physical and mental disabilities.

Characteristics of the family include household income, family compositions, country of birth of grandparents and the occurrence of 12 stressful life events within the previous year (moving to another address; a friend of the child moving to another address; tension at the parents’ work that has been felt at home; financial problems; rows with neighbors, friends, acquaintances or family; fire or burglary; problems with the physical health of people in close proximity; problems with the psychological health of people in close proximity; death of someone in close proximity; problems in the marriage relation; divorce and unemployment). If an event occurred, the perceived severity of the stress or tensions in the family caused by the event was additionally assessed.

### Received parenting support and use of care

In the baseline questionnaire, the use of various types of care in the previous year by the parent/caregiver, child and/or other children in the family will be assessed, including the general practitioner, medical specialists, psychological help, parenting support and other types of care (18 items). Furthermore, we include 3 items regarding visits to the youth health care centers.

In the follow-up measurement, the use of care will be assessed similarly as in the baseline questionnaire. Additionally, 3 items are included to assess whether parents/caregivers had questions or concerns regarding the upbringing of their child, whether they had been in need for parenting support and whether they have received parenting support during the follow-up period. Visits to youth health care centers, participation in parenting interventions, visits to parenting lectures and other types of parenting support during the last year are assessed by 20 items, including items considering the duration, frequency, character, content and the parents’/caregivers’ appreciation of the received parenting support. Additionally, when available and with permission of participants, data on received parenting support and care regarding parenting during the follow-up period will be collected from electronic files of preventive youth health care organizations.

### Elements in parenting interventions and other types of support

Elements of the parenting support that were received by parents/caregivers will be identified and coded. For this purpose, we will inventory the guidelines and/or protocols of the interventions that were reported by the parents/caregivers. In this study, content elements (objectives or general themes/principles) and delivery elements are extracted [12]. Delivery elements are operationalized as elements related to what was delivered in the intervention (i.e. the ‘active ingredients to change behavior’ or behavior change techniques) and how the intervention was delivered (i.e. who delivered, to whom, how often, for how long, in what format, and in what context). Behavior change techniques were categorized using the taxonomy of Michie [12]. This taxonomy includes 93 Behavior Change Techniques, grouped into 16 categories; for example the categories feedback and monitoring, comparison of behavior, repetition and substitution. Moreover, contextual (e.g. characteristics of target participants who receive the interventions) characteristics of the parenting interventions will also be assessed.

### Data management and statistical analysis

Data are handled according to the guidelines of the EU General Data Protection Regulation (GDPR). Data-management and analysis will be carried out at the Erasmus MC University Medical Center. Paper questionnaires will be transferred into electronic files and checked for missing or incorrect data. All data are handled confidentially and scientific data are stored anonymously.

Descriptive statistics will be used to describe the characteristics of the study population. We will use linear regression analysis for continuous outcome measures, and logistic regression analysis for dichotomous outcome variables. The exposure to (elements of) parenting interventions (yes/no) is the independent variable. In additional models, the baseline measurements and potential confounders (e.g. demographic characteristics of the caregivers and children) will be added. It will be evaluated whether additional multi-level regression analyses should be performed given the potential clustering within the youth health care organizations in the study. The exposure to parenting support will be based on information obtained from the follow-up questionnaire and when available the electronic files of the youth health care organizations. All analyses will be performed using SPSS version 24 for windows and R (version 3.4.1). Missing data will be handled by multiple imputation. All tests will be two-sided and *p*-values less than 0.05 will be considered as significant.

### Power considerations

In total, we aim to include net 1000 parents/caregivers in the study. With an expected 40% loss to drop-out between baseline and follow-up, we expect complete data of *n* = 600 participants. We assume that 50% (300) of these parents/caregivers will have received parenting support during the follow-up period. We assume an alpha of 0.05 (2-tailed) and power of 0.80. We apply a correction factor to account for the cluster design, assuming 25 clusters within the sample with an average cluster size of 24 participants and an intra-class correlation coefficient of 0.02. For this expected sample size and assumptions, with regard to the continuous outcome measures, a difference of 0.27 SD (standard deviation) between the subgroup exposed to parenting support and the subgroup not exposed to parenting support can be detected at follow-up. This is enough to indicate relevant effects, as a difference of 0.5 SD is considered meaningful [41, 42].

## Discussion

This article describes the study protocol of a prospective cohort study that is part of the CIKEO consortium study (Consortium Integration Knowledge promotion Effectiveness Of parenting interventions). The overarching aim of the CIKEO consortium is to investigate preventive parenting interventions, in order to identify effective elements within these interventions. The aim of the prospective cohort study is to investigate the exposure of parents/caregivers to parenting support elements and strategies in daily practice of Dutch preventive youth health care and the effects of this exposure on parenting skills, family functioning and child development in a naturalistic effect evaluation. We hypothesize that parents/caregivers who have been exposed to elements of parenting support during the follow-up period will show significant improvements in specific outcome variables related to parenting skills, family functioning and child development when compared to comparable parents/children who not have been exposed to parenting interventions.

This study has several strengths. First, the data are collected in a naturalistic setting, as the cohort study is conducted in the setting of daily youth health care practice. This enables us to explore the effects of parenting interventions in the “real world” rather than in an experimental setting, which will support the generalizability of our findings [19]. Furthermore, we will not only examine the effects of parenting interventions but also the effects of other types of parenting support provided by preventive youth health care. These types of parenting support are important to take into account, as previous research has indicated that such parenting support can also have relevant effects [43].

The proposed study also has some limitations and we expect to encounter some challenges. The first challenge of this study will be to include parents at risk of parenting problems. It may be a difficult group to reach, and these parents may be hesitant to participate in research [44]. To increase participation, we will recruit these parents through providers of parenting support. Second, because of the non-randomized design, results of the study are subject to confounding variables. We will assess the most important confounding variables; however, there may be residual confounding. Another limitation is that it is not possible to evaluate some general elements that have been shown to be of importance to the effectiveness of parenting interventions in our study design, such as the quality of the implementation of the interventions [7]. Although we cannot include it in our study, we know that these factors may be important to take into account and we emphasize the need to investigate these factors more thoroughly in future studies.

In conclusion, results of the naturalistic effect evaluation study will provide insight in which parenting support elements and strategies are used in daily practice, and which elements are effective. The results will be translated into products for practice and education; recommendations for practice and policy will also be made. The knowledge provided by CIKEO is valuable for various stakeholders in practice, including providers of parenting support (youth health care organizations and parent training institutes) and municipalities that provide interventions. It will enable professionals and policy makers to concentrate on elements of parenting support that are shown to be effective and to adapt parenting support to better fit the needs of clients and the possibilities within organizations and local settings. Furthermore, it may guide the future development and improvement of interventions. Ultimately, the goal is to make parenting support more flexible and more effective.

## Supplementary information

**Additional file 1: Table S1.** Interventions included by the CIKEO consortium.

## Data Availability

Data availability and sharing is non-applicable to this particular study as datasets are not yet generated nor analyzed for this study protocol. A data management plan and a checklist for processing data on individual persons is set up for this study. The dataset that will be generated and analysed for future publications, the data management plan and a checklist for processing data on individual persons is available from the corresponding author on reasonable request and with permission of the third parties involved.

## Abbreviations

BSI18: Brief Symptom Inventory 18
CBCL: Child Behavior Check List
CIKEO: Consortium Integration Knowledge promotion Effectiveness Of parenting interventions (the Dutch name: Consortium Integratie Kennisbevordering Effectiviteit Opvoedonzekerheid-interventies)
CJG: The Child and Family Centre (in Dutch: Centrum voor Jeugd en Gezin)
DSM: Diagnostic and Statistical Manual for Mental Disorders
FAD: Family Assessment Device
GDPR: EU General Data Protection Regulation
MSPSS: Multidimensional Scale of Perceived Social Support
PDH: Parenting Daily Hassles
PS: Parenting Scale
PSOC: Parenting Sense of Competence Scale
SD: Standard deviation

## References

[1] Oudhof M, M.S. de Wolff, M. de Ruiter, M. Kamphuis, L’Hoir MP, Prinsen B: Opvoedingsondersteuning voor hulp bij opvoedingsvragen en lichte opvoedproblemen (Parenting support for help with parenting questions and minor parenting problems). Utrecht, the Netherlands: Nederlands Centrum Jeugdgezondheid (Netherlands Expertcenter for Youth Health; NCJ); 2013.

[2] Zeijl E: Kinderen in Nederland (Children in the Netherlands). The Hague, the Netherlands: Sociaal en Cultureel Planbureau (Social and Cultural Planning Office); 2005.

[3] Reijneveld SA, de Meer G, Wiefferink CH, MR C (2008). Parents’ concerns about children are highly prevalent but often not confirmed by child doctors and nurses. BMC Public Health.

[4] Speetjens S, Van der Linden D, F G: Kennis over opvoeden: de vragen van ouders, het aanbod van de overheid en de mogelijkheden van de markt (Knowledge about parenting: the questions of parents, the offer of the government, and the possibilities of the market). Utrecht, the Netherlands: Trimbos-instituut (Trimbos Institute); 2009.

[5] Ince D: Wat werkt in Opvoedingsondersteuning? (What works in Parenting Support?). In*.* Utrecht, the Netherlands: Nederlands Jeugd Instituut (Netherlands Youth Institute; NJI) 2013.

[6] Moran P, Ghate D (2005). The effectiveness of parenting support. Child Soc.

[7] Moran P, Ghate D, Van Der Merwe A (2004). What works in parenting support? A review of the international evidence.

[8] Kaminski JW, Valle LA, Filene JH, CL B (2008). A meta-analytic review of components associated with parent training program effectiveness. J Abnorm Child Psychol.

[9] Sandler IN, Schoenfelder EN, Wolchik SA, DP M (2011). Long-term impact of prevention programs to promote effective parenting: lasting effects but uncertain processes. Annu Rev Psychol.

[10] Lundahl B, Risser HJ, MC L (2006). A meta-analysis of parent training: moderators and follow-up effects. Clin Psychol Rev.

[11] Bakermans-Kranenburg MJ, Van Ijzendoorn MH, F J (2003). Less is more: meta-analyses of sensitivity and attachment interventions in early childhood. Psychol Bull.

[12] Michie S, Richardson M, Johnston M, Abraham C, Francis J, Hardeman W, Eccles MP, Cane J, CE W (2013). The behavior change technique taxonomy (v1) of 93 hierarchically clustered techniques: building an international consensus for the reporting of behavior change interventions. Ann Behav Med.

[13] Chorpita BF, Daleiden EL (2009). Mapping evidence-based treatments for children and adolescents: application of the distillation and matching model to 615 treatments from 322 randomized trials. J Consult Clin Psychol.

[14] Leijten P, Gardner F, Melendez-Torres GJ, Hutchings J, Schulz S, Knerr W, G O (2019). Meta-analyses: key parenting program components for disruptive child behavior. J Am Acad Child Adolesc Psychiatry.

[15] van der Put CE, Assink M, Gubbels J, van Solinge NFB (2018). Identifying effective components of child maltreatment interventions: a meta-analysis. Clin Child Fam Psychol Rev.

[16] Molleman G, Fransen G (2012). Academic collaborative centres for health promotion in the Netherlands: building bridges between research, policy and practice. Fam Pract.

[17] Jansen MW, De Leeuw E, Hoeijmakers M, De Vries NK (2012). Working at the nexus between public health policy, practice and research. Dynamics of knowledge sharing in the Netherlands. Health research policy and systems.

[18] van Koperen MT, van der Kleij RM, Renders CC, Crone MM, Hendriks AM, Jansen MM, van de Gaar VV, Raat H, Ruiter EE, Molleman GG (2014). Design of CIAO, a research program to support the development of an integrated approach to prevent overweight and obesity in the Netherlands. BMC Obes.

[19] Kember D (2003). To Control or Not to Control: The question of whether experimental designs are appropriate for evaluating teaching innovations in higher education. Assess Eval High Educ.

[20] Biklen SK, Bogdan R (1986). On your own with naturalistic evaluation. New Directions for Program Evaluation.

[21] Verbrugge HP (1990). Youth health care in the Netherlands: a bird’s eye view. Pediatrics.

[22] Korfage IJ, Polder JJ, Koning HJ (2002). Time spent and costs of the clinics for youth health care. TSG-Tijdschrift voor Gezondheidswetenschappen.

[23] Horrevorts EM, van Grieken A, Broeren SM, Bannink R, Bouwmeester-Landweer MB, Hafkamp-de Groen E, Raat H (2015). Design of a controlled trial to evaluate the effectiveness of supportive parenting (‘Stevig Ouderschap’): an intervention to empower parents at increased risk of parenting problems by providing early home visits. BMC Psychol.

[24] P H: Generalist working with youth and families in the Netherlands. In*.*, vol. 2019. Utrecht, the Netherlands: Nederlands Jeugd Instituut (Netherlands Youth Institute; NJI) 2013.

[25] Zwikker M, Van Dale D, Kuunders M: Erkenningscommissie Interventies. Werkwijze en procedure (Committee for the Accreditation of Youth Interventions in the Netherlands; Methods and procedures). In*.* Utrecht, the Netherlands: Nederlands Jeugd Instituut (Netherlands Youth Institute; NJI), National Institute for Public Health and the Environment (RIVM); 2009.

[26] Wake M, Nicholson JM, Hardy P, Smith K (2007). Preschooler obesity and parenting styles of mothers and fathers: Australian national population study. Pediatrics.

[27] Paterson G, Sanson A (1999). The association of behavioural adjustment to temperament, parenting and family characteristics among 5-year-old children. Soc Dev.

[28] Arnold DS, O'leary SG, Wolff LS, Acker MM (1993). The parenting scale: a measure of dysfunctional parenting in discipline situations. Psychol Assess.

[29] Crnic KA, Booth CL (1991). Mothers’ and fathers’ perceptions of daily hassles of parenting across early childhood. J Marriage Fam.

[30] Crnic KA, Greenberg MT (1990). Minor parenting stresses with young children. Child Dev.

[31] Gibaud-Wallston J, Wandersmann LP: Development and utility of the parenting sense of competence scale in: the annual meeting of the American Psychological Association. Toronto, Canada; 1978.

[32] Byles J, Byrne C, Boyle MH, Offord DR (1988). Ontario child health study—reliability and validity of the general functioning subscale of the McMaster family assessment device. Fam Process.

[33] Epstein NB, Baldwin LM, Bishop DS (1983). The McMaster family assessment device. J Marital Fam Ther.

[34] Achenback TM, Rescorla LA (2000). Manual for the ASEBA preschool forms & profiles.

[35] Zimet GD, Dahlem NW, Zimet SG, Farley GK (1988). The multidimensional scale of perceived social support. J Pers Assess.

[36] Pedersen SS, Spinder H, Erdman RA, Denollet J (2009). Poor perceived social support in implantable Cardioverter defibrillator (ICD) patients and their partners: cross-validation of the multidimensional scale of perceived social support. Psychosomatics.

[37] Abdel-Khalek AM (2006). Measuring happiness with a single-item scale. Soc Behav Personal Int J.

[38] Ware JE, Kosinski M, Keller SD (1996). A 12-item short-form health survey: construction of scales and preliminary tests of reliability and validity. Med Care.

[39] Derogatis LR (2000). The brief symptom inventory–18 (BSI-18): administration, scoring and procedures manual.

[40] Landgraf JM, Abetz L, Ware JE (1996). The CHQ User’s manual.

[41] Cohen J (1977). Statistical power analysis for the behavioral sciences.

[42] Juniper EF, Guyatt GH, Willan A, Griffith LE (1994). Determining a minimal important change in a disease-specific quality of life questionnaire. J Clin Epidemiol.

[43] de Graaf I, Onrust S, Haverman M, Janssens J (2009). Helping families improve: an evaluation of two primary care approaches to parenting support in the Netherlands. Infant Child Dev.

[44] Landsmeer-Beker EA, Bouwmeester-Landweer MBR, Korbee-Haverhoek HD, Kousemaker NPJ, Baartman HEM, JM W (2006). Differences between respondents and non-respondents on a postal questionnaire addressing risk factors for child maltreatment.

