# Supplementary material for: Evaluation of effectiveness of (elements of) parenting support in daily practice of preventive youth health care; design of a naturalistic effect evaluation in ‘CIKEO’ (consortium integration knowledge promotion effectiveness of parenting interventions)
Source: BMC Public Health. 2019 Nov 6;19:1462. doi: 10.1186/s12889-019-7785-y (PMC6836651; doi:10.1186/s12889-019-7785-y)
Supplement: Supplementary file 1 — Additional file 1: Table S1. Interventions included by the CIKEO consortium. [file 12889_2019_7785_MOESM1_ESM.docx]

| Table S1: Interventions included by the CIKEO consortium |
| --- |
| **Name ( Translation)** |
| 1. Coach je kind (Coach your child) |
| 2. Gordon-training: Effectief communiceren met kinderen (Gordon training: Effective communication with children) |
| 3. Incredible Years (basis) |
| 4. Kortdurende Video-Hometraining (K-VHT) in gezinnen met jonge kinderen (Short-term Video Home Training (K-VHT) in families with young children) |
| 5. Video-hometraining (VHT) in gezinnen met kinderen in de basisschoolleeftijd (Video home training (VHT) in families with children of primary school age) |
| 6. Moeders Informeren Moeders (MIM) (Mothers Informing Mothers (MIM)) |
| 7. Opvoeden & zo (Educate & so) |
| 8. Pedagogisch adviseren (Pedagogical advice) |
| 9. Themis Opvoedcursus - Voor moeders uit niet-westerse migrantengroepen (Themis Parenting Course - For mothers from non-Western migrant groups) |
| 10. Triple P |
| 11. Video-feedback Intervention to Promote Positive Parenting and Sensitive Discipline (VIPP-SD) |
| 12. Praten met kinderen (Talking to children)* |
| 13. Peuter in Zicht (Toddler in Sight) |
| 14. Drukke kinderen (Hyperactive children) |
| 15. NIKA (NIKA) |
| 16. Stap voor stap 2 (Step by step 2) |
| 17. Stevig Ouderschap (Supportive Parenting) |
| 18. VoorZorg (Dutch version of Nurse-Family Partnership) |
| 19. Beter Omgaan met Pubers (Dealing with Teens )* |
| 20. Home-Start |
| 21. Shantala babymassage |

*These interventions will not be included in the cohort study, because they are targeted at parents/caregivers of children who are older than 7 years old. Details on these interventions can be found on the website of Dutch national Database of Effective Youth Interventions (www.nji.nl/nl/Databank/Databank-Effectieve-Jeugdinterventies)
